# An Evaluation of the Performance of Tag SNPs Derived from HapMap in a Caucasian Population

**DOI:** 10.1371/journal.pgen.0020027

**Published:** 2006-03-10

**Authors:** Alexandre Montpetit, Mari Nelis, Philippe Laflamme, Reedik Magi, Xiayi Ke, Maido Remm, Lon Cardon, Thomas J Hudson, Andres Metspalu

**Affiliations:** 1 McGill University and Genome Quebec Innovation Centre, Montreal, Quebec, Canada; 2 Institute of Molecular and Cell Biology of the University of Tartu, Tartu, Estonia,; 3 Estonian Biocentre, Tartu, Estonia; 4 Wellcome Trust Centre for Human Genetics, University of Oxford, Oxford, United Kingdom; 5 The Estonian Genome Project Foundation, Tartu, Estonia; University of Alabama at Birmingham, United States of America

## Abstract

The Haplotype Map (HapMap) project recently generated genotype data for more than 1 million single-nucleotide polymorphisms (SNPs) in four population samples. The main application of the data is in the selection of tag single-nucleotide polymorphisms (tSNPs) to use in association studies. The usefulness of this selection process needs to be verified in populations outside those used for the HapMap project. In addition, it is not known how well the data represent the general population, as only 90–120 chromosomes were used for each population and since the genotyped SNPs were selected so as to have high frequencies. In this study, we analyzed more than 1,000 individuals from Estonia. The population of this northern European country has been influenced by many different waves of migrations from Europe and Russia. We genotyped 1,536 randomly selected SNPs from two 500-kbp ENCODE regions on Chromosome 2. We observed that the tSNPs selected from the CEPH (Centre d'Etude du Polymorphisme Humain) from Utah (CEU) HapMap samples (derived from US residents with northern and western European ancestry) captured most of the variation in the Estonia sample. (Between 90% and 95% of the SNPs with a minor allele frequency of more than 5% have an *r* ^2^ of at least 0.8 with one of the CEU tSNPs.) Using the reverse approach, tags selected from the Estonia sample could almost equally well describe the CEU sample. Finally, we observed that the sample size, the allelic frequency, and the SNP density in the dataset used to select the tags each have important effects on the tagging performance. Overall, our study supports the use of HapMap data in other Caucasian populations, but the SNP density and the bias towards high-frequency SNPs have to be taken into account when designing association studies.

## Synopsis

The recent completion of the Haplotype Map (HapMap) project of the human genome provides considerable information on the patterns of variation in the genome of four populations. One of the applications is a description of a set of tags that act as proxies for many other surrounding variants. This will greatly help researchers in their quest to find complex disease genes by reducing the number of genetic variants to test in association studies. To evaluate its usefulness, several aspects of the map, including its transferability to other populations, still needed to be verified experimentally. Using genomic regions where variants had been thoroughly documented in Caucasian samples from Estonia, the researchers found that the transferability of tags is extremely good. The researchers also found that variants with low frequency in the general population (i.e., less than 5%) could not be accurately captured with tags, and that the regional density of variants in the HapMap project had a major impact on the performance of the tags. This research indicates that the HapMap project will be useful, but that careful consideration of hypotheses and study design will be essential for the success of association studies.

## Introduction

The main objective of the HapMap project is to provide the research community with a description of the linkage disequilibrium (LD) structure of the human genome in order to enable the optimization of the single-nucleotide polymorphism (SNP) selection process for association studies [1]. Many algorithms have been described that can minimize the number of SNPs required (i.e., tag single-nucleotide polymorphisms [tSNPs]) to adequately represent the genetic variation across a specific region (or the entire genome). Early algorithms were mostly based on the concept of common haplotypes within haplotype blocks (haplotype tag single-nucleotide polymorphisms [htSNPs]) [2–7]. However, there are many limitations to these algorithms as the haplotype block boundaries vary with the SNP density used and also between sample sets, making it difficult to compare and adapt the approach to different populations [8,9]. In addition, extensive LD can exist between adjacent blocks, introducing some redundancy in the htSNP set. Today, the most commonly used tagging algorithms employ only the LD properties of the SNPs (or haplotypes), such as *r* ^2^, which is entirely independent of the haplotype block concept [10–12]. Furthermore, since *r* ^2^ with the disease variant is inversely proportional to the increase in sample size required to achieve comparable power to detect it, the use of those algorithms facilitates the study design.

The four population samples used in the HapMap project were proposed as references for tSNP selection in other world populations. However, it is not known how well the information extracted using the HapMap project will work in different populations. Another factor that can affect the success of tSNP selection and, consequently, the association studies themselves is the fact that the first phase of the HapMap project aimed to obtain genotypes for one common SNP (minor allele frequency [MAF] > 5%) per 5 kbp [13]. This bias towards common SNPs and the relatively sparse spacing could result in an important loss of information even in a closely related sample set. Finally, since the size of each study sample used to create the HapMap project varied from 90–120 independent chromosomes, it is not clear whether this sample size is large enough to capture and convey all the useful information.

**Figure 1:**
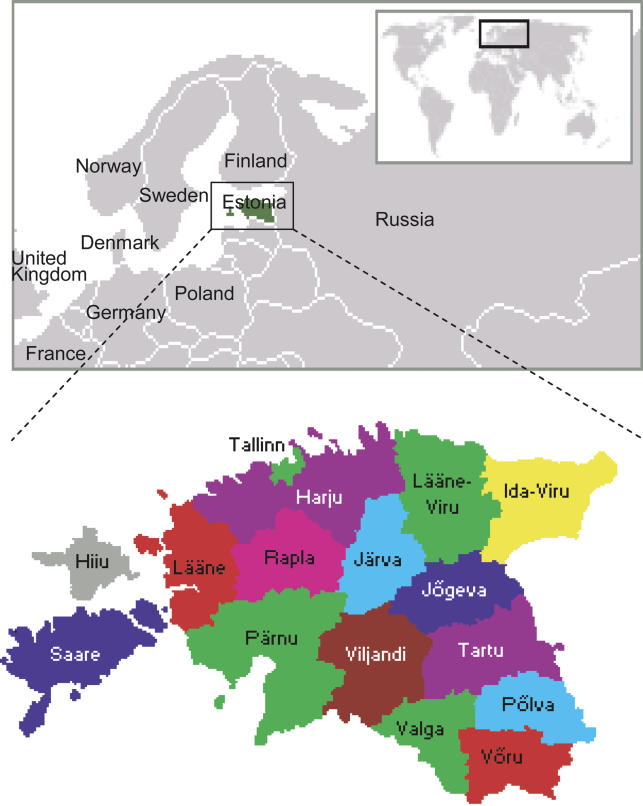
Map of Estonia

This report aims to describe the transferability and performance of tSNPs selected from the HapMap project. To do this, 1,090 individuals from all over Estonia, a country that has been influenced by many different migrations from Europe and Russia, were studied (Figure 1). We selected SNPs from two 500-kbp ENCODE regions (http://www.genome.gov/10005107) on Chromosome 2 with very different recombination rates that have been resequenced entirely in 48 individuals from various origins and in which all SNPs have been genotyped in four populations as part of the HapMap project.

## Results

### MAF Distribution

**Figure 2:**
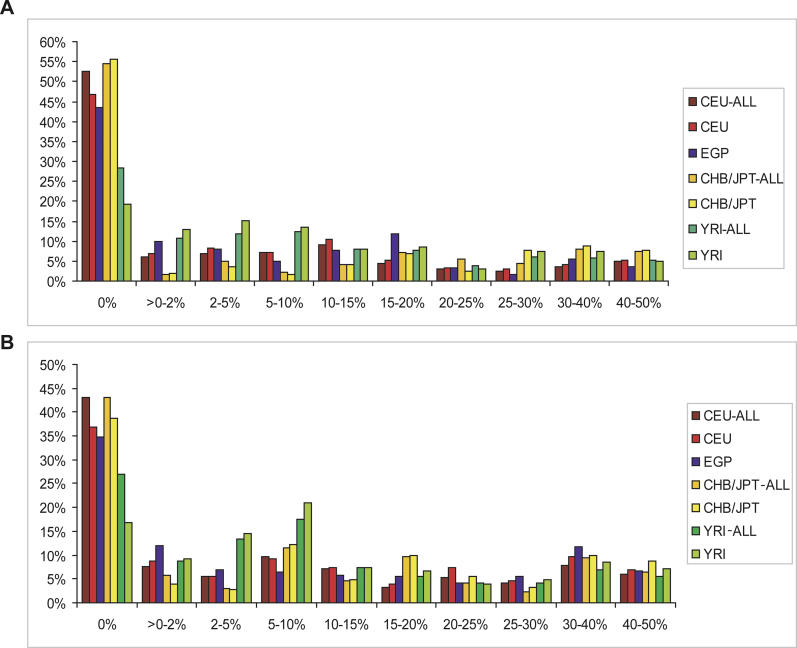
Distribution of Allelic Frequency of the Selected SNPs (A) Distribution in the ENCODE 1 (2p16.3) region. (B) Distribution in the ENCODE 2 (2q37.1) region. The -ALL groups refer to the entire set of markers typed in the HapMap project.

Two 500-kbp ENCODE regions on Chromosome 2 were selected for this study: ENr112 on 2p16.3 (ENCODE 1) and ENr131 on 2p37.1 (ENCODE 2). These two regions have previously been resequenced in their entirety in 48 individuals and all SNPs genotyped as part of the HapMap project. The regions have different average recombination rates (0.8 cM/Mbp for ENCODE 1 and 2.1 cM/Mbp for ENCODE 2). Overall, there are 2,431 and 2,067 SNPs in ENCODE 1 and ENCODE 2, respectively, that have been successfully genotyped as part of the HapMap project. In each of two 500-kbp ENCODE regions, 768 random SNPs were selected and genotyped in 1,090 samples from the Estonian Genome Project (EGP). In ENCODE 1 and ENCODE 2, 721 and 699 SNPs, respectively, passed all genotyping quality criteria and were used for the remainder of the study. Allele frequencies of genotyped SNPs were compared to frequencies of all SNPs in the ENCODE regions (Figure 2).

With the exception of a small reduction in the number of monomorphic SNPs for the CEPH (Centre d'Etude du Polymorphisme Humain) from Utah (CEU) and Yoruba from Ibadan, Nigeria (YRI) samples (owing to a lower representation of those SNPs in the original dataset [see Materials and Methods]), we did not observe a marked bias in the distribution of the selected SNP allele frequencies. Also, we observed that the proportion of low-frequency SNPs in the Asian samples (the Chinese from Beijing [CHB] and Japanese from Tokyo [JPT] samples were analyzed together throughout this study) is much lower than in the other samples, particularly in the ENCODE 1 region. This is probably due to the history of these populations and not because of a bias in the original SNP-discovery process. [13] Overall, the distribution of allele frequencies for the EGP sample appears to be similar to that for the CEU sample.

### LD and Population Structure

**Figure 3:**
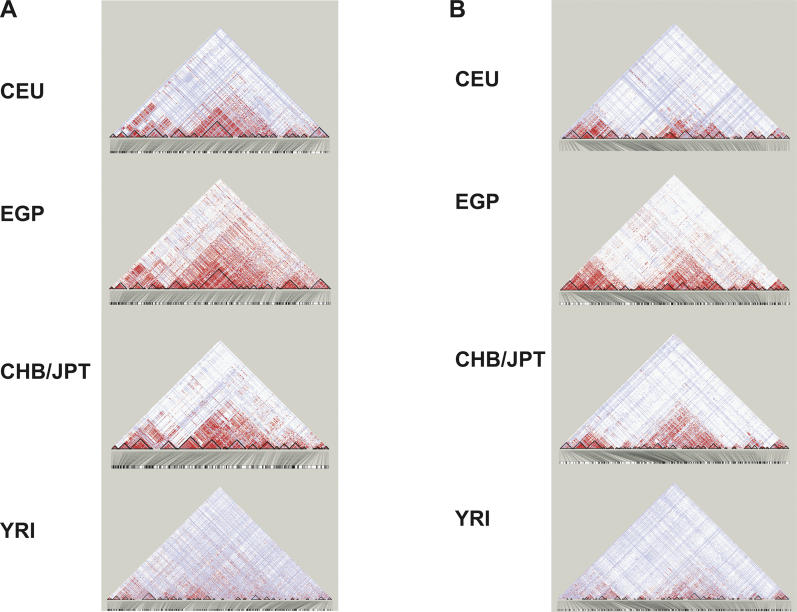
LD/Block Structure of the HapMap and Estonia Samples (A) ENCODE 1 region. (B) ENCODE 2 region.

**Figure 4:**
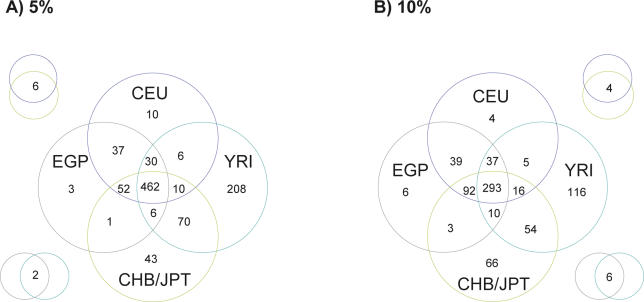
Four-Way Comparison of Common SNPs The Venn diagram shows the number of shared common SNPs using (A) 5% or (B) 10% as the MAF threshold. For clarity, extra circles for areas not captured in the main diagram are shown.

Figure 3 shows the LD structure for both ENCODE regions in the EGP and in the three HapMap samples. For both regions, the LD structure appears to be well-conserved across all non-African samples. Overall, LD is lower in the ENCODE 2 region, which is consistent with the fact that its recombination rate is almost three times as high as the ENCODE 1 region. Although the block distribution in the EGP closely resembles the CEU distribution, its LD structure appears to match the CHB/JPT sample better. A four-way comparison of common alleles is presented in Figure 4. As expected, the YRI sample contains the majority of population-specific common alleles. It also shows that more than 95% of the common SNPs in either the EGP or CEU are also common in the other Caucasian sample.

A two-way comparison of allele frequencies is presented in Figure S1. Since the presence of Asian-specific chromosomes in the Estonia population could be masked when looking only at SNP frequencies, we performed median-joining network analyses (Figures S2 and S3). This network method has been mostly used so far for human mitochondrial DNA and Y-chromosome data analysis in phylogenetic studies [14]. As expected, we observed that the EGP sample usually shares its haplotypes with the CEU. However, our results also showed that, depending on the region analyzed, the two Caucasian samples can have different haplotype frequencies and that some haplotypes are seen only in the EGP sample or are shared with the CHB/JPT sample. Finally, by using the Structure program (http://pritch.bsd.uchicago.edu/structure.html), we could not detect any population substructure within Estonia since the likelihood increased continuously with increasing *K* values and since the assignment of the individuals in the *K* populations was equivalent for each of the 14 counties.

### tSNPs

**Figure 5:**
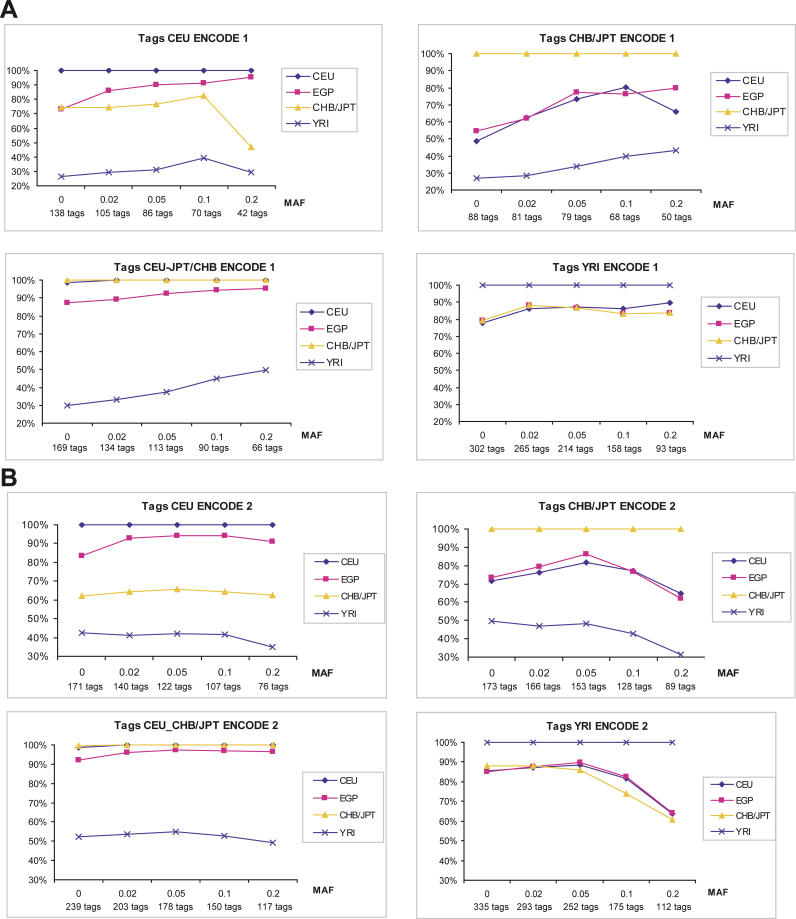
Performance of Tags Selected from HapMap Samples Tags were selected from one or two HapMap samples, and the performance plotted was measured in the indicated population (A) in the ENCODE 1 region and (B) in the ENCODE 2 region. Only polymorphic SNPs with at least the specified MAF were used to select either the tags or to calculate the performance. The number of tags used for each MAF studied is indicated at the bottom of each graph.

To verify the transferability of tags across populations, we selected tSNPs at various allele frequencies from a HapMap sample and measured their performance in all the other population samples. The pairwise algorithm of the Tagger program was used to select the tags. These are selected to represent all untagged SNPs with a high correlation coefficient [12]. An *r* ^2^ of 0.8 was defined as the coefficient threshold for tag selection and performance measurement. Figure 5 shows the performance of tSNPs selected from the CEU, CHB/JPT, and YRI populations. CEU tags perform equally well in the EGP sample in both ENCODE regions, but it must be noted that many more tags were needed in the lower LD region. More than 90% of the SNPs were correlated with an *r* ^2^ of more than 0.8 for all SNPs with a MAF of 5% or more.

A similar observation was made with the reverse strategy by using tags from the EGP sample (unpublished data). The CHB/JPT tags, on the other hand, performed less well on the CEU or EGP samples, usually capturing less than 80% of the SNPs. Overall, in the ENCODE 1 region, tags selected to have a minimum MAF of 10% showed the best performance at capturing SNPs in any population, while in the ENCODE 2 region, it was tags selected to have a minimum MAF of 5% that showed the best performance. The YRI tags worked surprisingly well in all the samples, but at the expense of using two to three times more tSNPs. In many instances, we observed that the tagging performance drops sharply for SNPs with a MAF of 10% or more. This is due to the presence of many SNPs with high allelic frequency in the target population, but with frequencies lower than the selected MAF threshold in the population used to select tags. As expected, this effect was more pronounced when using very divergent population samples. A mimimal tSNP set generated from the combination of tags from both the CEU and CHB/JPT samples improved the tagging performance of EGP SNPs by about 2%–5% in both ENCODE regions, but also with an increase of about 20%–30% in the number of tSNPs used.

### Effect of MAF

**Figure 6:**
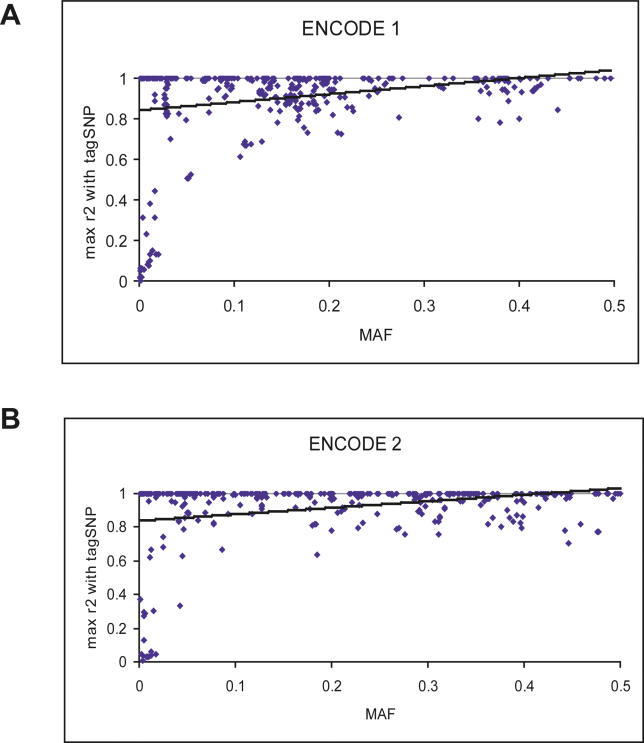
Maximum Distribution (*r* ^2^) of SNPs from Estonia in Relation to CEU tSNPs Tags were selected from all polymorphic SNPs of the CEU population in (A) the ENCODE 1 region (138 tSNPs) and (B) the ENCODE 2 region (171 tSNPs).

The effect of varying the MAF parameter for SNP selection is best exemplified in Figure 6, which shows the maximum *r* ^2^ of all EGP SNPs against CEU tSNPs plotted as a function of the MAF. As expected, markers with more MAFs in the EGP sample tend to correlate better with tSNPs from the CEU population. An important aspect illustrated by Figure 6 is that markers with a MAF of less than 5% are poorly captured by CEU tSNPs. From these low-MAF markers, 17% in the ENCODE 1 region and 23% in the ENCODE 2 region do not have an *r* ^2^ of more than 0.5 with any of the tSNPs. On the other hand, all of the high-frequency markers (MAF > 20%) are correlated to a tag with an *r* ^2^ of more than 0.7, a value that would still be highly useful for association studies.

### Sample Size

**Figure 7:**
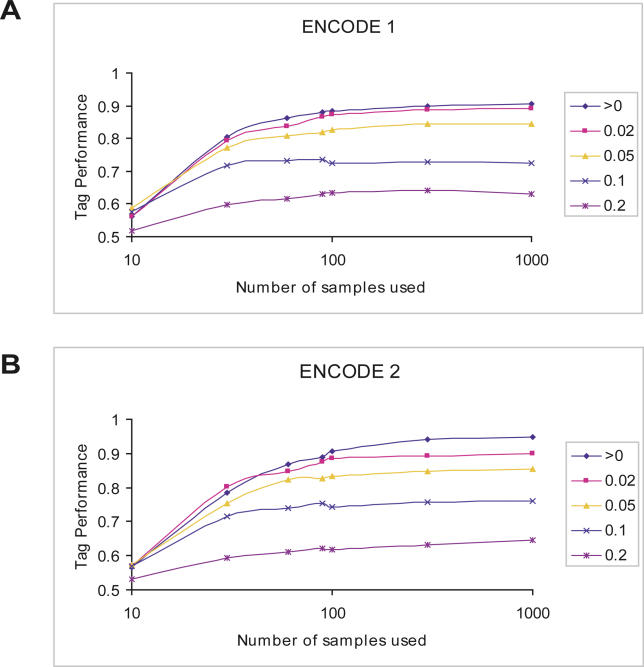
Effect of Sample Size on Tagging Performance Random sets of 10, 30, 60, 100, 300, and 1,000 EGP samples were used to select tags at different MAF thresholds (shown as different colored lines). Tags were then tested in the CEU population, and the ratio of tagged versus all polymorphic SNPs (using an *r* ^2^ threshold of 0.8) was plotted for (A) the ENCODE 1 region and (B) the ENCODE 2 region. An average of 100 tests is shown.

The effect of the sample size used to derive the tags was verified using random sets of ten to 1,000 EGP samples. For each dataset, an average of 100 tests was used to evaluate the performance of the tags relative to all polymorphic SNPs on the CEU sample (Figure 7). Overall, as expected, we observed that the sample-size effect was more important for less-frequent SNPs (less than 5%). At MAFs of 5% or more, optimal tagging is obtained with about 90–100 independent samples. However, the difference in tagging performance using a sample size of 60 was non-significant for these SNPs.

### Density Effect

**Figure 8:**
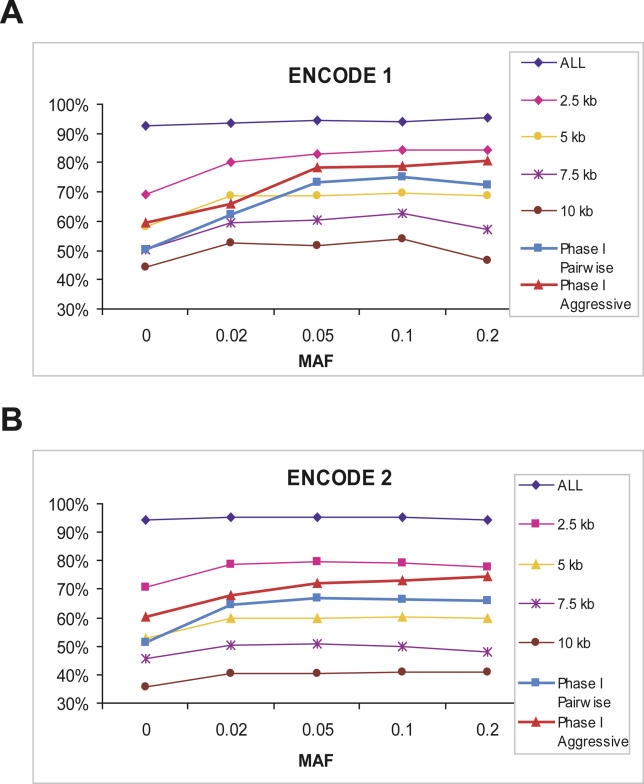
Effect of SNP Density on Tag Selection Tags were selected from the CEU samples using random sets of SNPs averaging the specified densities. The ALL set contains all SNPs and corresponds to a density of one SNP every 1.3 kbp. The Phase I pairwise and aggressive sets contain only SNPs with a minimum MAF of 5% in the CEU sample, and tags were selected with the pairwise and aggressive algorithm of Tagger, respectively. The tagging performance was calculated on the EGP cohort by measuring the ratio of tagged SNPs over all polymorphic SNPs with at least the specified MAF for (A) the ENCODE 1 region and (B) the ENCODE 2 region.

The importance of the SNP density for tag selection was assessed using six different datasets. The 500-kbp ENCODE regions were divided into equal-sized windows, and one polymorphic SNP in the CEU population was selected in each. tSNPs were picked and their performances were measured in the EGP population. The ALL set includes all polymorphic SNPs and is equivalent to a density of about one SNP every 1.3 kbp. A clear drop in the tagging performance is observed for each decreasing density studied (Figure 8). The effect is more pronounced in the lower LD ENCODE 2 region. With the aggressive algorithm of Tagger (which uses both multimarker and pairwise LD), a clear improvement in the tagging efficiency was observed (unpublished data). At the highest density, tagging performance was not significantly affected, but there was an observed reduction of 10%–20% in the number of tSNPs required. At lower densities, the performance did improve with the use of multimarker proxies, and was up by as much as 15% for the 10-kbp density set of the ENCODE 2 region. Finally, in two cases (Phase I pairwise and Phase I aggressive), the selected SNPs were required to have a frequency of at least 5% so as to mimic the current Phase I of the HapMap project. While it shows that it improves the tagging performance of common SNPs as expected, only 65%–75% of these could be adequately tagged compared to more than 80% when using higher densities to select the tags.

## Discussion

In this study, we used a population sample with mixed European ancestry to evaluate the usefulness of the HapMap project. Phase I of the HapMap project, involving the genotyping of one frequent SNP every 5 kbp in each of four population samples, has recently been completed [13]. The HapMap project promises to deliver an easy-to-use tool that will facilitate association studies in any population by minimizing the number of SNPs to be genotyped. It is important to understand that several assumptions underlie this prediction. First, the SNP-selection process for the HapMap project was based on the common variant/common disease hypothesis, which states that common variants are responsible for common disease [15,16]. While many variants responsible for common diseases have recently been identified using LD mapping and tend to confirm the hypothesis [17], other possibilities such as rare variants and allelic heterogeneity would complicate or invalidate the use of the HapMap project [18].

In addition, several different tagging algorithms have been published, all producing different outcomes [19]. Most of the block-free algorithms use the *r* ^2^ measure to define how well a SNP can be a proxy for another SNP. An assumption behind this is that if SNP A is in high LD with SNP B and SNP B is in high LD with the true disease variant C, then A should be in high LD with the disease variant. Then, it can be shown that the increase in the number of samples necessary to achieve a specific power to detect the disease variant is proportional to the inverse of *r* ^2^ (reviewed in [18]). However, because of allelic heterogeneity, phenotype heterogeneity, population heterogeneity, or other confounding factors, it will not necessarily always be the case [20]. On the other hand, algorithms that use blocks to define tSNPs have other flaws including floating boundaries and tSNP redundancy [8,9].

With a popular program that uses *r* ^2^ to define a minimal set of tSNPs, we showed that tags derived from the CEU HapMap sample can be used to capture accurately the variation observed in samples from Estonia. Since this country has been settled by many migrations from Europe and Russia, it shows that the HapMap project will be useful in many, if not all, other Caucasian populations, with the possible exception of population isolates. The approach of using two populations to select a minimal tagging set (B. N. Howie et al., unpublished data), in this case the CEU and CHB/JPT samples, can improve tagging performance. Such a strategy could be used when the population ancestry cannot be entirely assigned to one HapMap group.

Since the SNPs used in this study were selected from ENCODE regions previously analyzed exhaustively for their DNA variation content, and because no selection bias was observed, the data obtained in this study should closely reflect reality. Using the pairwise algorithm, the number of tSNPs necessary to essentially cover the genome has been evaluated at 600,000 or one tag/5 kbp in the CEU population using the ENCODE regions [13]. In the low-recombination ENCODE 1 region, one tag every 6 kbp was needed to capture all common alleles (>5% frequency), while one tag every 4 kbp was needed in the high-recombination ENCODE 2 region. Even though those regions differ widely from each other in their recombination rate and LD structure, when complete information was present to select the tags, no significant difference in the tagging performance in the Estonia sample was observed. However, the applicability of these conclusions to the entire genome will need a sampling of more regions.

One parameter that had not been verified experimentally for the HapMap project is the effect of the sample size to select the tSNPs (the HapMap CEU and YRI plates each contained 60 independent samples, while the CHB/JPT plate contained 89 independent samples). Our study clearly indicates that optimal performance is obtained with about 90 independent samples. However, for common SNPs (>5% frequency), the use of 60 independent samples did not affect significantly the performance of tags.

The parameter that has the biggest impact on tag selection is the SNP density. With a density of one common SNP every 5 kbp, which corresponds to Phase I of the HapMap project, it has been estimated that about 75% of the genome would be covered adequately by tags in the CEU sample using the pairwise algorithm of Tagger and an *r* ^2^ of 0.8 as a correlation threshold [13]. This is approximately what we observed with our dataset, but increasing the density improved significantly the fraction of SNPs captured. As much as 20% more SNPs were captured in the low-LD ENCODE 2 region each time the density increased by a factor of two (up to one SNP every 1.3 kbp). The use of multimarker tags (i.e., the aggressive algorithm of Tagger) does improve the efficiency by reducing the number of tags required to obtain similar results. An improvement in the performance was observed when sparser sets (i.e., less than one SNP every 1.3 kbp) were used to derive the tags, indicating that the use of this aggressive algorithm would be advantageous in association studies. However, more analyses need to be done to verify how well multimarker tests are transferable between populations (i.e., if the same haplotypes are always predicting the same markers) and how much the performance is affected by missing data.

This exercise shows that the SNP density used for selection of tSNPs has a major effect and indicates that Phase I of the HapMap project is probably not optimal for tag selection. Moreover, because of the existing bias in the current databases for SNPs with a high frequency and because the sample sizes used are not large enough to describe exhaustively the less common alleles, one can see that tag selection and hence disease-association studies with rare variants (i.e., less than 5%) will always be inefficient. Phase II of the HapMap project, which aims to genotype approximately 3 million additional SNPs, should increase the density by about 3- to 4-fold (to about one SNP/1.3 kbp). However, the density of common SNPs in Phase II of the HapMap project will not be uniform throughout the genome, which will potentially affect the tagging performance, particularly in low-LD regions.

Other studies have also attempted to measure the transferability of tSNPs across non-HapMap European samples [21–23]. The main conclusions drawn from these reports are that, generally, tags derived from the HapMap project can be transferred with little loss of performance to other populations. Similar observations have been made with samples of Asian or African-American origins (P. de Bakker, personal communication). The few examples where suboptimal tagging performance was observed in these studies could be explained by poor SNP coverage in a low-LD region or because of a bias in SNP selection.

In conclusion, the HapMap project will be useful for association studies in many different populations. Phase II of the HapMap project, which recently added 3 million SNPs to the existing map, bringing it to a density of roughly one SNP every kbp, will improve the tagging performance substantially. However, exhaustive tagging may not be possible for low-frequency SNPs or in regions of very low LD because not all regions of the genome will receive equivalent coverage. Finally, SNP-ascertainment bias, allelic heterogeneity, and study design will need to be further considered to accurately evaluate the usefulness of the HapMap project to identify disease variants.

## Materials and Methods

### Population samples.

The 1,090 DNA samples used in this study were selected from 10,317 samples of the Biobank of the EGP Foundation (EGP samples). Eighty samples (40 males and 40 females) were selected randomly, according to the place of birth, from each of 13 Estonian counties (Harju, Ida-Viru, Jõgeva, Järva, Lääne-Viru, Põlva, Pärnu, Rapla, Saaremaa, Tartu, Valga, Viljandi, Võru), and 50 samples (25 males and 25 females) were selected from the combined Hiiumaa and Läänemaa counties (Figure 1). Prior to collection, we obtained approval from the Ethics Committee of the EGP Foundation and informed consent from all participating subjects. The CEU, YRI, CHB, and JPT samples were genotyped as part of the HapMap project.

### SNP selection and genotyping.

The two 500-kbp ENCODE regions on Chromosome 2 are ENr112 (ENCODE 1: NCBI Build 34 [http://www.ncbi.nlm.nih.gov/genome/guide/mouse/contig/Build34.html] positions 51633239–52133238) and ENr131 (ENCODE 2: NCBI Build 34 positions 234778639–235278638). From each region, 768 SNPs genotyped at the McGill University and Genome Quebec Innovation Centre as part of the HapMap project were selected out of 2,180 and 1,893 SNPs, respectively. SNPs that failed to give reliable genotyping assays in all three HapMap samples (CHB and JPT populations were combined) were not included in this selection process. The total number of monomorphic SNPs in all populations included in the selection process was set at 100 for each region (out of 226 for ENCODE 1 and 209 for ENCODE 2). At the time of SNP selection, 251 SNPs in ENCODE 1 and 174 SNPs in ENCODE 2 had not yet been genotyped by the HapMap project. We therefore also included these SNPs in the selection process. Genotyping was performed on an Illumina Bead Laboratory platform (http://www.illumina.com) as previously described [24]. Genotyping was successful in 1,054 of the 1,090 samples. Successful assays were obtained with 721 SNPs in the ENCODE 1 region and 699 SNPs in the ENCODE 2 region in all populations (i.e., having a call rate of more than 80%, being in Hardy-Weinberg equilibrium, and having no more than one Mendelian transmission or reproducibility error) and were used for the remainder of the study (Tables S1 and S2). No reproducibility error was observed in the Estonia panel out of 46 replicate samples that were distributed across all plates used in the study.

### Population structure.

LD measures and haplotype block structure were obtained with the program Haploview (http://www.broad.mit.edu/mpg/haploview) using the confidence-interval method [3]. The haplotype structure of the four populations (EGP, CEU, CHB/JPT, and YRI) was analyzed using phylogenetic networks. For each haploblock, median-joining networks connecting the haplotypes with frequencies >1% from 60 randomly chosen individuals from each population were generated using Network 4.1.1.1 (http://www.fluxus-engineering.com) [14]. The substructure of the Estonian population was tested with the Structure program [25]. We used an admixtured ancestry model with 10,000 burning steps and 10,000 Markov-chain Monte Carlo steps. The number of possible subpopulations *(K)* was tested from 1 to 20. Each test was repeated three times.

### Selection of tSNPs and performance tests.

tSNPs were selected from the subset of SNPs with a MAF equal to or greater than the studied MAF in a reference population using the pairwise or the aggressive option of the Haploview version of the Tagger program (http://www.broad.mit.edu/mpg/haploview), which is an extension of the algorithm developed by Carlson et al. [12]. An *r* ^2^ of 0.8 was selected as a threshold for all analyses. Performance was defined as the number of SNPs in the evaluated population that have an *r* ^2^ of more than 0.8 with the tSNPs over the total number of SNPs considered. All performance measures consist of an average of ten tests to account for the intrinsic randomness of the algorithm. MultiPop-TagSelect was applied to select tSNPs from the combined CEU/CHB–JPT population (B. N. Howie et al., unpublished data). Basically, this procedure involves selecting the maximally informative set of SNPs from the combined set of each population's tSNPs. To test the effect of SNP density, each ENCODE region was divided into windows of equal size (corresponding to the desired density) and one polymorphic SNP was randomly selected from each. To mimic the HapMap Phase I data, one SNP in every 5-kbp window was selected, with a frequency of at least 5%. tSNPs were then picked from the resulting dataset and the performance was measured as described above. All measurements were repeated ten times.

To evaluate the effect of the SNP MAF, a tSNP set derived from all polymophic SNPs was obtained for both ENCODE regions using the CEU population sample according to the *r* ^2^-bin method with an *r* ^2^ cutoff of 0.8. For each marker tested in the EGP sample, we calculated the CEU tSNP showing the highest *r* ^2^ score. To measure the effect of sample size, random datasets of individuals from the EGP sample were selected and tSNP sets were obtained as described above at different MAF cutoffs. These tagging sets were tested on the CEU sample and the performance was measured on all polymorphic markers. Sampling and testing from each dataset was performed 100 times and the average was calculated.

## Supporting Information

Figure S1 Two-Way Correlation of Allelic Frequencies Allelic frequencies for each of the 1,420 SNPs ordered by their position on Chromosome 2 were compared between (A) EGP and CEU samples, (B) EGP and CHB/JPT samples, and (C) EGP and YRI samples.
(1.7 MB TIF)

Figure S2 Examples of Median-Joining Haplotype Networks in the ENCODE 1 Region A total of 60 individuals from each of the four population samples were used. Median-joining haplotypes are shown for three haplotype blocks in the ENCODE 1 region. (A) Twenty-four SNPs from a 14-kbp block (Chr2 51644128–51658569); (B) 103 SNPs from a 96-kbp block (Chr2 51808385–51904698); and (C) 35 SNPs from a 25-kbp block (Chr2 52010053–52034659).
(3.7 MB TIF)

Figure S3 Examples of Median-Joining Haplotype Networks in the ENCODE 2 Region A total of 60 individuals from each of the four population samples were used. The networks are shown for three haplotype blocks in the ENCODE 2 region. (A) Fifty-four SNPs from a 71-kbp block (Chr2 234795506–234867063); (B) 48 SNPs from a 35-kbp block (Chr2 235126155–235161153); and (C) 32 SNPs from a 19-kbp block (Chr2 235201671–235221211).
(3.4 MB TIF)

Table S1 List of Selected SNPs and Their Frequency in All Population Samples for the ENCODE 1 Region (172 KB XLS)

Table S2 List of Selected SNPs and Their Frequency in All Population Samples for the ENCODE 2 Region (173 KB XLS)

## Abbreviations

CEU: CEPH (Centre d'Etude du Polymorphisme Humain) from Utah
CHB: Chinese from Beijing
EGP: Estonian Genome Project
HapMap: Haplotype Map
htSNP: haplotype tag single-nucleotide polymorphism
JPT: Japanese from Tokyo
LD: linkage disequilibrium
MAF: minor allele frequency
SNP: single-nucleotide polymorphism
tSNP: tag single-nucleotide polymorphism
YRI: Yoruba from Ibadan, Nigeria
